# A256 INCIDENCE OF CIRRHOSIS AMONG RECENT IMMIGRANTS AND REFUGEES TO ONTARIO, CANADA : A POPULATION-BASED COHORT STUDY

**DOI:** 10.1093/jcag/gwac036.256

**Published:** 2023-03-07

**Authors:** N Sayed, N Selzner, M Djerboua, H Muaddi, P Neves, J Flemming

**Affiliations:** 1 Medicine, Queen's University, Kingston; 2 Medicine, University of Toronto, Toronto; 3 ICES, Queen's University, Kingston; 4 Surgery, University of Toronto; 5 Centre for Living Donation, University Health Network, Toronto; 6 Public Health Sciences, Queen's University, Kingston, Canada

## Abstract

**Background:**

The burden of cirrhosis is increasing in North America where a significant proportion of the population (~20%) is comprised of recent immigrants and refugees. However, the incidence of cirrhosis among this population has not been described.

**Purpose:**

The aim of this study was to describe the incidence of cirrhosis among recent immigrants and refugees to Ontario, Canada stratified by cirrhosis etiology and region of origin and compare incidence rates to those of Canadian-born and long-term residents.

**Method:**

This is a population-based cohort study from Ontario, Canada from 2000-2017 using healthcare data housed at ICES. Individuals with incident cirrhosis using a 5-year lookback were identified along with cirrhosis etiology using validated algorithms. Recent immigrants and refugees to Ontario (since 1985) and their World Bank region of origin were defined based on linkage to the Immigration, Refugees, and Citizenship Canada’s Permanent Resident database. Adjusted incidence rates of cirrhosis by immigration status, etiology, and region of origin were calculated and differences in rates were compared between recent immigrants and refugees to long-term residents (born in Canada or arriving before 1985) using age and sex adjusted Poisson regression to calculate rate ratios (RR).

**Result(s):**

In total, n=25,054 recent immigrants/refugees with incident cirrhosis were identified. 59% were male, median age was 49 years (IQR 39-61), 99% resided in urban areas and 31% resided in neighbourhoods of the lowest income quintile. The most common regions of origin were East Asia and Pacific (n=8,121; 32%), South Asia (n=5,576; 22%), Europe and Central Asia (n=4,454; 18%) and Latin America/Caribbean (n=3,216; 13%). The majority had NAFLD cirrhosis (n=13,972; 55%), followed by HBV (n=4,648; 19%), ALD (n=2,957; 12%), and HCV (n=2,635; 11%). Cirrhosis incidence stratified by etiology varied by region of origin, with HBV highest among the East Asia and Pacific and Sub-Saharan Africa cohort (Figure 1b), HCV among Sub-Saharan Africa and South Asia (Figure 1c), ALD among European/Central Asia and South Asia (Figure 1d), and NAFLD among the Latin America/Caribbean, Middle East/North Africa, and South Asian populations (Figure 1e). When comparing age and sex adjusted incidence of cirrhosis by etiology and immigration status, rates were lower among recent immigrants/refugees for all causes compared to long-term residents (all RR <1.0; Figure 1a,c-f) with the exception of HBV where it was over 4-fold higher (Figure 1b).

**Image:**

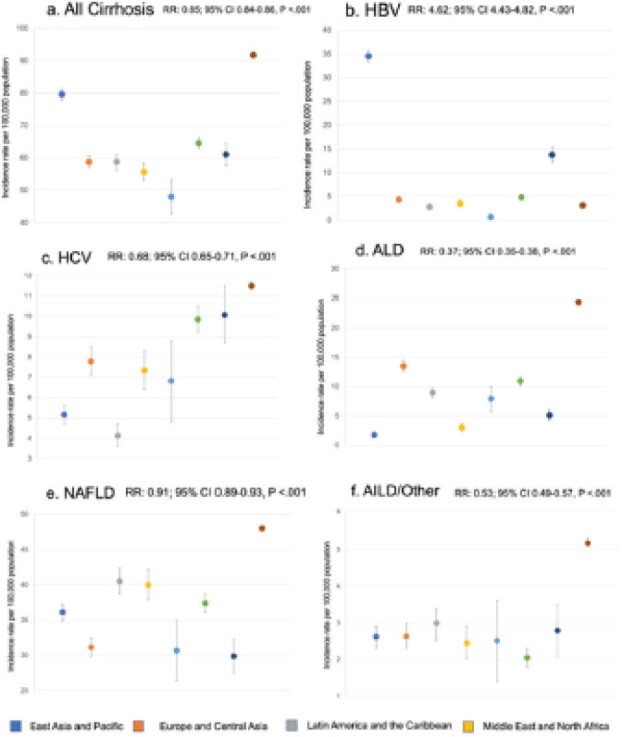

**Conclusion(s):**

Rates of HBV cirrhosis are substantially higher among migrants to Ontario while rates of all other causes of cirrhosis are lower, likely explained by the healthy immigrant effect. NAFLD has emerged as the most common etiology of cirrhosis among migrants with rates approaching those of Canadian-born and long-term residents. These data add to our understanding of the evolving epidemiology of cirrhosis and the contribution of other etiologies outside of viral hepatitis among immigrants.

**Please acknowledge all funding agencies by checking the applicable boxes below:**

None

**Disclosure of Interest:**

None Declared

